# The current practice of aspiration prophylaxis in obstetric anesthesia: a survey among non-physician anesthetic providers working in hospitals in Ethiopia

**DOI:** 10.1186/s12871-021-01478-4

**Published:** 2021-10-26

**Authors:** Metages Hunie, Efrem Fenta, Simegnew Kibret, Diriba Teshome

**Affiliations:** Department of Anesthesia, School of Medicine, College of Health Sciences, Debre Tabor University, PO. Box: 272, Debre Tabor, Ethiopia

**Keywords:** Obstetric anesthesia, Aspiration prophylaxis, Anesthetic providers, Ethiopia

## Abstract

**Background:**

Pulmonary aspiration is one of the most important complications of obstetric anesthesia. Prevention of pulmonary aspiration is commonly performed by the application of different anesthetic maneuvers and administration of drugs. This study aimed to assess the non-physician anesthetic providers current practice of aspiration prophylaxis during anesthesia for cesarean section in Ethiopia.

**Methods:**

This survey study was conducted from October 01 to November 05, 2020, on a total of 490 anesthetic providers working in hospitals in Ethiopia. A structured checklist was used to collect data from non-physician anesthetic providers.

**Results:**

Four hundred and ninety (490) anesthetic providers participated in our study. The majority of the respondents (84%) were working in the public sector. Most of the cesarean delivery was done under regional anesthesia and more than half of anesthetic providers in Ethiopia administered aspiration prophylaxis routinely. Metoclopramide was the most frequently given as a prophylaxis for pulmonary aspiration.

**Conclusions:**

More than half of the anesthetic providers administered aspiration prophylaxis routinely. Metoclopramide was the commonest administered aspiration prophylaxis for parturients who underwent cesarean delivery to prevent aspiration.

**Supplementary Information:**

The online version contains supplementary material available at 10.1186/s12871-021-01478-4.

## Introduction

Cesarean section (CS) was introduced in clinical practice as a life-saving procedure both for the mother and the baby [1]. Currently, most CS is done under regional anesthesia techniques [2, 3]. It has not without a public health concern as it is associated with morbidity and mortality [4].

In Ethiopia, the prevalence of C/S is higher than [5, 6] the World Health Organization recommended rate which is up to 15% [7]. Studies conducted in Ethiopia revealed that about 30% of cesarean deliveries were developed complications after anesthesia [8] and others studies indicated that over half of cesarean deliveries were performed under general anesthesia [9, 10].

According to the American College of Obstetricians and Gynecologists (ACOG) report, cesarean delivery significantly increased woman’s risk of pregnancy-related morbidity and mortality which accounts (35.9 deaths per 100,000 live deliveries) as compared to a woman having vaginal delivery (9.2 deaths per 100,000 live births) [11].

Even though the mortality rate of pulmonary aspiration of gastric contents has declined, it is one of the most important complications of general anesthesia in obstetric patients [1, 12, 13]. Increased risk of aspiration is due to prolonged gastric emptying time in labor, increased intra-abdominal pressure due to the gravid uterus, and relaxation of the lower esophageal sphincter due to hormonal changes [14–16].

To reduce this risk numerous measures and maneuvers are used to prevent aspiration of acid gastric contents during general anesthesia (GA) [17–20]. The morbidity and mortality of this complication can be significantly reduced by decreasing the acidity of the inhaled contents. These include preoperative fasting, non-particulate antacids, H2 receptor blockers, gastro kinetic drugs like metoclopramide, rapid sequence induction with cricoid pressure, and awake extubation during emergence from general anesthesia [21–23].

Pulmonary aspiration could lead to poor patient outcomes once it occurs. Prevention is paramount important in medicine. Its role is more pronounced in a resource-limited setting; when the cost of medical care is highly lacking. This study might be baseline information for further researchers and might be supportive information for the scientific world. This study aimed to assess the anesthetic providers current practice of aspiration prophylaxis during anesthesia for cesarean section in Ethiopia.

## Methods

### Study setting, design, period, and population

There are ten geographical regions and two city administrations in Ethiopia. A total of 490 anesthesia professionals working in hospitals of Ethiopia were included in this survey from October 01 to November 05, 2020. Anesthetic providers in Ethiopia can be physician or non-physician anesthetic providers. Non-physician anesthetic providers include Master of Science in anesthesia who are trained for 2 years after graduating with a Bachelor of Science degree in anesthesia, Bachelor of Science degree in anesthesia who are trained 4 years of university training or 3 years of additional training after accomplishing nursing diploma, and Level V anesthetic providers who trained a diploma nurse, and additional one-year anesthesia training. In Ethiopia, almost all anesthesia service is covered by non-physician anesthetic providers. This study was conducted only in non-physician anesthetic providers.

### Sampling technique

All available non-physician anesthetic providers working in Hospitals of Ethiopia were surveyed.

### Data collection technique

A structured checklist regarding the current practice of anesthesia on aspiration prophylaxis for CS was used to collect data. This tool for data collection was adopted from ASA and Perinatology guidelines [21]. The data collection tool has two subsections; section one socio-demographic variables (Age, Sex, region, etc.), and anesthetic providers practice of aspiration prophylaxis for Obstetric anesthesia (anesthetic maneuvers, drugs for aspiration prophylaxis, etc.).

A questionnaire was constructed using a google form and the link (https://forms.gle/nCQtvSnqYjcm49us5) was sent to all non-physician anesthetic providers working in Ethiopian hospitals through the common telegram group and individual email address to get a better response rate. The Telegram Messenger (Telegram Inc. Dubai UAE; www.telegram.org) group has 730 anesthetic providers.

### Data analysis

Data were checked manually for completeness and then coded by using the SPSS (Statistical Package for Social Sciences/Statistical Product and Service Solution (IBM Corp. Armonk NY USA) version 23 computer program for analysis. Descriptive statistics were employed to summarize the results.

### Data quality control

The investigators, cross-checked for the completeness, and consistency of the data before data analysis.

## Results

### Socio-demographic characteristics of the respondents

Four hundred and ninety non-physician anesthetic providers have participated with a response rate of 67%. The majority of the respondents (84%) were working in the public sector (Table 1).

**Table 1:** Socio-demographic characteristics of the Respondents

Variables	Frequency	Percentage
Age (mean)	–	–
29 ± 7 year		
Sex		
Male	353	72
Female	137	28
The educational level of anesthetic providers		
Level V	29	6
BSc	281	57
MSc	180	37
Anesthesia Working experience		
< 5 years	257	52
5–10 years	218	45
> 10 years	15	3
Anesthetic providers working Hospitals		
Public Sector	409	84
Private Sector	12	2
Public and Private Sector	69	14
The Level of hospitals		
Primary	76	15
General	172	35
Referral	170	35
University teaching hospitals	72	15

*Note*: *BSc* Bachelor of Science degree, *MSc* Master of Science degree

### The practice of anesthetic providers for aspiration prophylaxis

**Fig. 1:**
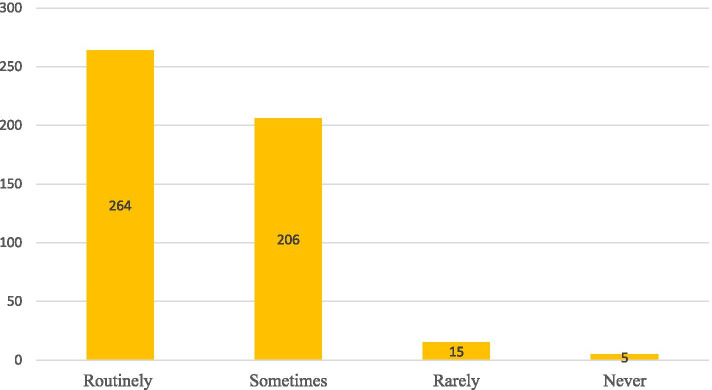
Frequency of aspiration prophylaxis use among anesthetic providers who are working in hospitals of Ethiopia

**Fig. 2:**
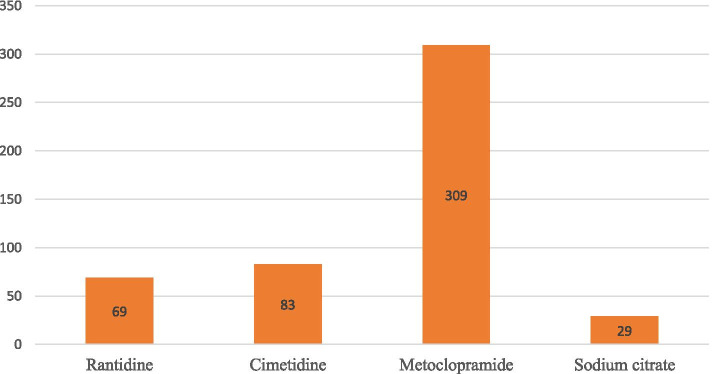
Commonly drugs used for aspiration prophylaxis use among anesthetic providers who are working in hospitals of Ethiopia

More than half of the anesthetic providers were administered aspiration prophylaxis routinely. Metoclopramide was the most commonly administered as a prophylaxis for pulmonary aspiration (Table 2, Figs. 1 and 2).

**Table 2:** The practice of anesthetic providers for aspiration prophylaxis

Variables	Frequency	Percentage
Anesthesia technique		
Spinal Anesthesia	461	94
General Anesthesia	29	6
Induction technique for GA?		
Modified RSI	228	46
RSI	262	54
Use of cricoid pressure		
Yes	455	93
No	35	7
NPO for clear fluids		
2–3 h	472	96
6–8 h	18	4
NPO for solids		
2–3 h	17	4
6–8 h	473	96
Do you have an aspiration prophylaxis protocol for parturients		
Yes	283	58
No	207	42
level of risk of aspiration for parturients		
high risk	463	95
Low risk	27	5
Do you use more than one drug for the prevention of aspiration?		
Yes	366	75
No	124	25
Extubation Techniques		
Deep	18	4
Awake	472	96
Do you anticipate a policy change soon?		
Yes	382	78
No	108	22

## Discussion

In Ethiopia, about 30% of mothers who underwent cesarean deliveries were developed complications after anesthesia [8]. Studies conducted in others settings of Ethiopia indicated that over half of cesarean deliveries were performed under general anesthesia [9, 10] that might increase aspiration-related maternal morbidity and mortality. While Aspiration is a commonly reported complication during Cesarian delivery globally; we do not have any specific data on its prevalence in Ethiopia. This risk might be minimized by the use of aspiration prophylaxis [12, 24, 25]. Actions taken to prevent aspiration of gastric contents may depend on the assessment of the level of risk of aspiration; administration of drugs; and application of different anesthetic maneuvers (e.g., RSI) are common strategies of prevention [19, 20, 26].

Administering preoperative gastrointestinal stimulants, gastric acid secretion blockers, and antacids might be used for patients at increased risk of pulmonary aspiration. Routine administration of preoperative gastrointestinal stimulants, gastric acid secretion blockers, and antacids to reduce the risk of pulmonary aspiration in patients with no apparent increased risk for pulmonary aspiration is not recommended (Table 3) [24, 27–33].

**Table 3:** A review of currently used drugs for aspiration prophylaxis

S.No	Drugs Used for Aspiration Prophylaxis	Current recommendations
1	Gastrointestinal Stimulants (Metoclopramide)	• Admnistering preoperative gastrointestinal Stimulants might be used for patients at increased risk of pulmonary aspiration. • Routine administration of preoperative gastrointestinal stimulants for the purpose of reducing the risk of pulmonary aspiration in patients with no apparent increased risk for pulmonary aspiration is not recommended.
2	Gastric Acid Secretion blockers (Proton pump inhibitor: omeprazole, pantoprazole; Histamine-2 receptor antagonists: cimetidine, ranitidine;)	• Administering drugs that block gastric acid secretion preoperatively may be used in patients at increased risk of pulmonary aspiration. • Routine administration of preoperative gastric acid secretion blockers for the purpose of reducing the risk of pulmonary aspiration in patients with no apparent increased risk for pulmonary aspiration is not recommended.
3	Antacids (sodium citrate)	• Administering nonparticulate antacids preoperatively may be used in patients at increased risk of pulmonary aspiration. • Routine administration of preoperative non-particulate antacids to reduce the risk of pulmonary aspiration in patients with no apparent increased risk for pulmonary aspiration is not recommended.

Our study showed that the majority of the respondents (94%) perform spinal anesthesia for cesarean delivery which is in line with a study done in Israel as 95% of the cases are done under regional anesthesia [34]. This finding is dissimilar with research done in Turk by Mehmet Aksoy et al. on anesthesia techniques for cesarean sections as the proportion of general anesthesia was about 45% which is too high as compared to our finding and this discrepancy could be justified by their study is a retrospective analysis of last decade data [35].

The finding of this study indicated that more than half of anesthetic providers (54%) administered aspiration prophylaxis routinely. While a survey study of UK obstetric unit on acid aspiration prophylaxis in labor found that an increase in the use of acid aspiration prophylaxis for at risk parturients to 61% [36]. This discrepancy might be due to clinical setup differences as the UK is the most developed country and they might have clinical evidence-based clinical practice guidelines.

The current study showed 93% of anesthetists used cricoid pressure with rapid sequence induction, and about 96% of patients were extubated fully awake. Similarly, a study done in England by Desai N et al. on a survey of the practice of rapid sequence induction for cesarean section found that cricoid pressure is applied for 98% of the cases [37] and Shaikh et al. showed that 84% of anesthetic providers used rapid sequence induction with cricoid pressure during general anesthesia, while about 50% of anesthetic providers performed extubation when patients were fully awake. In contrast to our findings, antacids were used by 90% of the anesthetic providers [23]. This difference may be due to the limited availability of antacids.

Our study showed that most of the anesthetic providers working in hospitals of Ethiopia give metoclopramide (63%) followed by Cimetidine (17%), ranitidine (13%), and sodium citrate (6%) for parturients who underwent CS delivery to prevent aspiration. In contrast to our finding, a study conducted in New Zealand by Kluger et al. showed that 47% of anesthetic providers administered metoclopramide, 72% of anesthetic providers give H2 antagonists, and 95% of anesthetic providers administered sodium citrate as prophylaxis for pulmonary aspiration [38]. Another study done in England by Desai N et al. on a survey of the practice of rapid sequence induction for cesarean section found that metoclopramide, ranitidine, and sodium citrate were used for 43, 86, and 88% of the case to prevent aspiration [37]. A difference in a clinical setting could be a probable justification for this discrepancy.

## Conclusions

More than half of the anesthetic providers administered aspiration prophylaxis routinely. Metoclopramide was the commonest administered aspiration prophylaxis for parturients who underwent CS delivery to prevent pulmonary aspiration. Prevention is paramount important in medicine. Its role is more pronounced in resource-limited settings; when the cost of medical care is highly lacking.

## Limitation

The limitation of this study might be we only surveyed non-physicians anesthetic providers, no data on pulmonary aspiration risk or prevalence in our settings, lack of national protocols for prevention of pulmonary aspiration.

## Supplementary Information

**Additional file 1.**

## Data Availability

The datasets used and/or analyzed during the current study are available from the corresponding author on reasonable request.

## Abbreviations

ACOG: American College of Obstetricians and Gynecologists
CS: Cesarean Section
GA: General Anesthesia
RA: Regional anesthesia
RSI: Rapid sequence induction
WHO: World Health Organization
