# Supplementary material for: The current practice of aspiration prophylaxis in obstetric anesthesia: a survey among non-physician anesthetic providers working in hospitals in Ethiopia
Source: BMC Anesthesiol. 2021 Oct 26;21:256. doi: 10.1186/s12871-021-01478-4 (PMC8549307; doi:10.1186/s12871-021-01478-4)
Supplement: Supplementary file 1 — Additional file 1. [file 12871_2021_1478_MOESM1_ESM.docx]

Socio-demographic characteristics

1. Age ----------------------------

2. Sex

A. Male

B. Female

3. Educational level of anesthetist’s

A. level V ----------------

B. BSc --------------------

C. MSc ----------------------------

4. Anesthesia Working experience in years: -----------------------

5. Anesthetists working at:

A. Public Sector Hospitals ---------------

B. Private Sector Hospitals --------------

C. Public and Private Sector Hospitals--------------

6. The Level of hospitals

A. primary

B. general

C. Referral

D. university teaching hospitals

7. Which anesthesia technique is commonly used in your practice for cesarean section

A. Regional Anesthesia (RA)

B. General Anesthesia (GA)

8. Which induction technique is used for GA?

A. modified RSI

B. RSI

9. Is Cricoid Pressure applied

A. Yes

B. No

10. NPO for clear fluids

A. 2–3 hours

B. 6–8 hours

11. NPO for solids

A. 2–3 hours

B. 6–8 hours

12. How frequently do you administer prophylaxis for acid aspiration?

A. Routinely to all women

B. Sometimes

C. Never

D. Rarely

13. Do you categories parturient into levels of risk of aspiration?

A. Yes

B. No

14. If yes Questioner # 13 which group parturient are considered

A. high risk

B. Low risk

15. Which aspiration prophylaxis you commonly used in your setting?

A. Ranitidine

B. Cimetidine

C. Metoclopramide

D. PPI

16. Do you use more than one drug for the prevention of aspiration?

A. Yes

B. No

17. Which Extubation techniques commonly used cesarean section under GA

A. Deep

B. Awake
